# Personality traits and BMI trends over three years in Japanese university students

**DOI:** 10.1371/journal.pone.0248833

**Published:** 2021-03-22

**Authors:** Nozomi Fukuhara-Makiyama, Masaki Hayashida, Masakazu Kobayashi, Ikuko Sagara, Sayaka Ogawa, Mayumi Maeda, Susumu Shirabe

**Affiliations:** 1 Unit of Preventive Medicine, Graduate School of Biomedical Sciences, Nagasaki University, Nagasaki, Japan; 2 Center for Health and Community Medicine, Nagasaki University, Nagasaki, Japan; University of Lleida, SPAIN

## Abstract

In Europe and America, associations between personality traits and body-mass index (BMI) have been reported. However, in Japan, the association between personality traits and BMI (i.e., thinness and obesity) has not been well studied. In this study, we investigated the relationship between Temperament and Character Inventory (TCI) personality traits and changes in BMI status among Japanese students during their university attendance. We measured the height and weight of 5,340 students in a Japanese university during annual medical checkups and calculated their BMI. The students’ personality traits were measured using the short Japanese version of the TCI at university admission. The participants were divided into seven groups based on how BMI changed from the first year to the fourth year at university. In men, compared to the group that maintained normal BMI status (*N* = 2,189) over time (i.e., the control group), the group that maintained thinness status (*N* = 226) were lower in Reward Dependence, and the group whose status improved from thinness to normal (*N* = 117) were higher in Harm Avoidance. In women, compared with the control group (*N* = 1,510), the group that maintained thinness status (*N* = 302) was lower in Novelty Seeking, and the group whose status worsened from normal to thinness (*N* = 127) was higher in Harm Avoidance. Weak associations were found between thinness and TCI personality traits among Japanese university students. Further elaboration of the relationship between obesity or thinness and personality traits may help to provide effective preventive interventions in these areas.

## Introduction

Both obesity and thinness are known to have detrimental effects on physical health. Obesity is related to increasing risks of other lifestyle-related diseases including heart disorder, cerebrovascular disease, diabetes, and arteriosclerosis. Additionally, thinness is related to increasing risks of undernutrition, amenorrhea, osteoporosis, and osteopenia [1].

In Japan, the prevalence of obesity and/or thinness has been increasing among university students, based on the Japanese definitions of obesity as a body-mass index (BMI) ≥ 25 kg/m^2^ and thinness as a BMI < 18.5 kg/m^2^. According to a 2015 report on National University students published by the Japanese National University Council of Health Administration, the prevalence of both thinness and obesity increased among male university students as compared to the prevalence found in the equivalent report published in 2000. Among female university students, the prevalence of obesity increased, while that of thinness decreased [2]. Furthermore, university students are more likely to consume less fruits and vegetables, consume more high-fat foods, live a sedentary lifestyle, smoke, consume alcohol and engage in other unhealthy lifestyles [3–7]. There are also reports that physical activity decreases while in school [5, 8–10]. The years from 18 to 25 are an important transition period characterized by the absence of parental supervision. Therefore, students must display autonomy in decision-making [11] while likely experiencing significant changes in their living environment. Thus, such students might be more likely to experience obesity and/or thinness, compared to middle- and high-school students, because the former tend to have more difficulty in maintaining healthy lifestyles [12]. However, as these unhealthy behaviors can continue throughout adulthood [13], interventions for university students are essential in this context.

Previous research has shown that personal factors contribute greatly to an individual’s life management in general, including weight control. In Europe and the United States, personality traits are among the factors found to be related to BMI as an index of obesity [14, 15]. Therefore, it is important to measure and consider an individual’s personality traits to provide effective and continuous interventions to prevent obesity and thinness.

Regarding personality measurement, Cloninger’s Temperament and Character Inventory (TCI) is widely used [14, 16, 17]. The TCI is an instrument that assesses both temperament and character traits [18, 19]. The former are the four dimensions of *Novelty Seeking* (NS; i.e., behavioral activation), *Harm Avoidance* (HA; i.e., behavioral inhibition), *Reward Dependence* (RD; i.e., behavioral adjustment), and *Persistence* (P; i.e., behavioral maintenance), while the latter are the three dimensions of *Self-Directedness* (SD; i.e., self-control), *Cooperativeness* (C; i.e., cooperation), and *Self-Transcendence* (ST; i.e., spirituality). The temperament traits are considered to be influenced by genetic factors, while the character traits are considered to be influenced by environmental factors. Moreover, obesity and thinness should be examined not only in terms of individuals’ genetic factors but also in terms of environmental factors.

The obesity rates of Japan differ greatly from those of Europe and the United States. Based on the international criteria to define obesity (i.e., a BMI ≥ 30 kg/m^2^), the obesity rates in Japan are 4.8% for men and 3.7% for women, while those in Europe and the United States are at least 20% and 35%, respectively, for both men and women [20]. Therefore, the results of previous studies on the relationship between personality traits and obesity and/or thinness conducted in Europe and the United States [21] are not directly applicable to Japanese people. Additionally, few studies of these relationships involved Japanese participants. Furthermore, many studies of the association between TCI personality traits and BMI were cross-sectional. In one longitudinal study, the researchers simply analyzed the relationship between personality traits and changes in BMI levels over time; however, no studies to date have specifically analyzed changes in terms of which groups of people with normal BMI became obese or thin over time [22].

Therefore, our primary interest in this study was to investigate if there are differences in TCI personality traits between those who maintain a normal BMI status over time and those who do not maintain such a status among Japanese university students during their university attendance.

## Methods

### Participants

**Fig 1:**
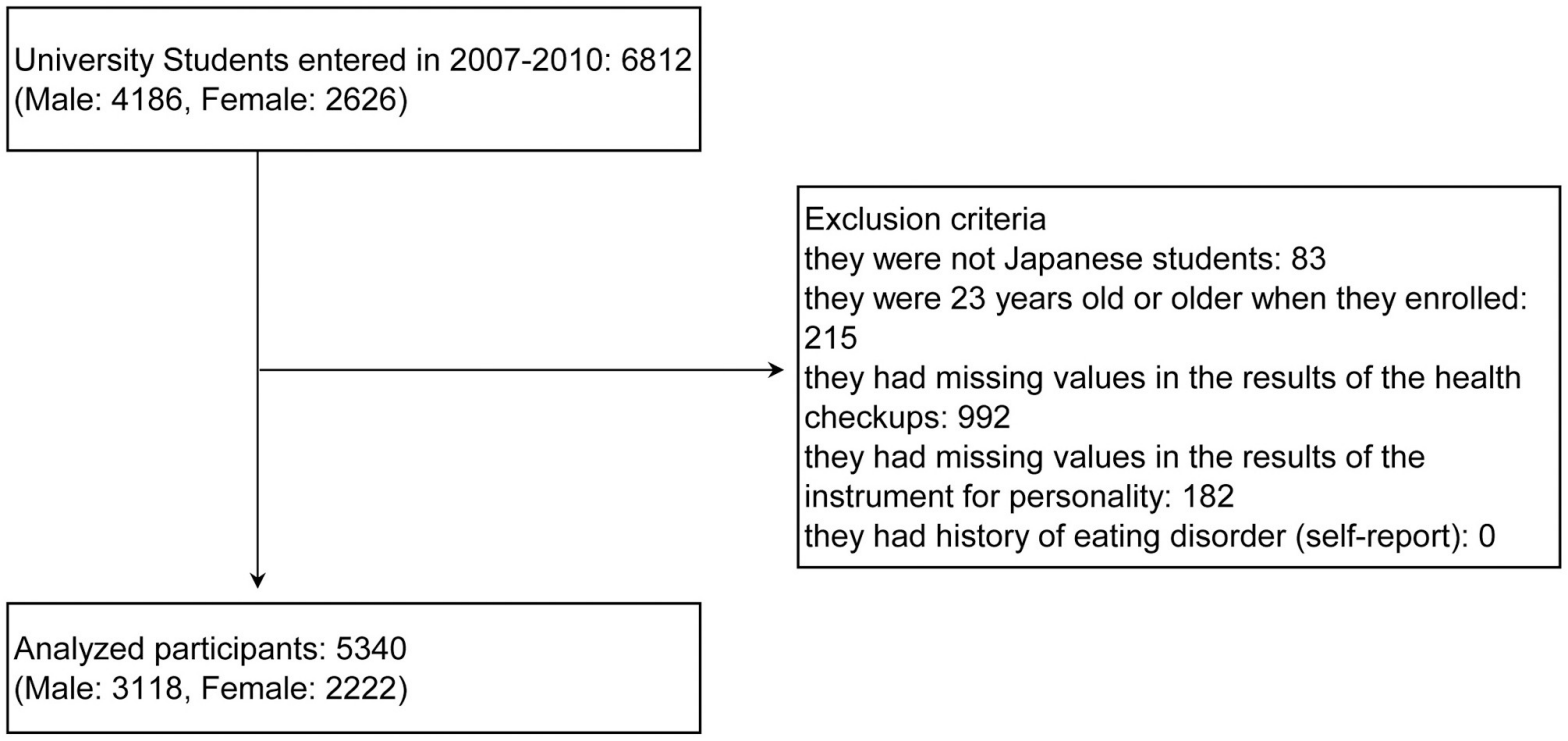
Flowchart of the analysis participants selection.

This longitudinal study initially included 6,812 university students (4,186 men) who entered the university in 2007. Participants were excluded if they were not Japanese students (83), or if they were aged 23 or older (215) when they enrolled (i.e., each participant’s age was required to be within 1 SD of the mean age of all enrolled students). Furthermore, they were excluded if they had any missing values in the results of the health checkups (992) or the personality measure (182), or if they had a history of eating disorders (self-report) (0). As a result, the data from a total of 5,340 (3,118 men) participants who did not meet the exclusion criteria were used for the analyses (Fig 1). This final set of participants had no missing values, and no outliers were observed for the main variables of this study. Informed consent was obtained in written form from all participants.

### Measures

At the time of the annual health checkups, height and weight were measured, and BMI was calculated. The TANITA TBF202 body composition meter with an automatic height meter was used (Tanita Inc., Tokyo, Japan). We measured the participants’ personality traits using the Japanese short version (125 items) of the TCI (two-point scale; i.e., yes/no), when they entered the university.

### Statistical analysis

All values of continuous variables were summarized as means and standard deviations. The student’s unpaired *t*-test was used to compare differences in means between men and women for each variable.

We defined thinness as BMI < 18.5 kg/m^2^, normal as 18.5 kg/m^2^ ≤ BMI < 25 kg/ m^2^, and obesity as BMI ≥ 25 kg/m^2^ based on the criteria of the Guidelines for the Management of Obesity Disease 2016 [23]. Based on these three categories of the BMI level (i.e., normal, obesity, and thinness), participants were classified into seven groups depending on how their status, in terms of these categories, changed from the first year to the fourth year. The seven groups were Thinness-to-Thinness (TT), Thinness-to-Normal (TN), Normal-to-Thinness (NT), Normal-to-Normal (NN), Normal-to-Obesity (NO), Obesity-to-Normal (ON), and Obesity-to-Obesity (OO).

*T*-scores for each TCI personality trait among the seven groups were calculated and compared by one-way analysis of variance (ANOVA). *T*-scores transform the raw scores to adjust them to a distribution where the mean score is 50 and the standard deviation is 10. The distribution indicates the number of standard deviations of the results from the mean. Using the NN group as the control, Dunnett’s test was used to conduct multiple comparisons of the means of *t*-scores of the other groups to those of the control group. These analyses were conducted separately for men and women.

The significance threshold was set at *p* < .05 (two-tailed) for all the analyses including the ANOVA and Dunnett’s test. SPSS ver. 23 software (SPSS Inc., Chicago, IL, USA) was used for all analyses.

### Ethical issues

This study followed the Declaration of Helsinki and the Ethical Guidelines for Medical and Health Research Involving Human Subjects of Japan (http://www.lifescience.mext.go.jp/files/pdf/n1500_01.pdf). This was an observational study that analyzed the values from students’ health checkups conducted annually at Nagasaki University as well as the values from the personality tests implemented at the time of admission. As this study did not require additional invasive procedures and interventions, neither written nor verbal informed consent to use their data for analyses were obtained from the study participants. However, “the matters to be disclosed regarding the implementation of such research when not receiving informed consent,” as indicated in the ethical guidelines for medical research targeting humans, were disclosed. To give participants the opportunity to refuse participation in this study, opt out materials were presented at university admission, and the data of participants who refused participation were deleted from the analyses. Information was stored in the health checkup database at Nagasaki University. All study protocols including the informed consent process were approved by the Institutional Review Board of Nagasaki University (approval no. 20022801–2).

## Results

Table 1 shows the demographic information of the participants in the present study. The mean age (standard deviation) of all the participants was 18.2 (0.5), and the mean BMI (standard deviation) was 21.1 kg/m^2^ (2.8). There were statistically significant differences between men and women in height, weight, BMI, and all TCI personality traits.

**Table 1:** Demographics information of the participants in this study.

	All participants		Male		Female	
Number	5340		3118		2222	
Age	18.2	(0.5)	18.3	(0.6)	18.2	(0.5)
Height	165.6	(8.4)	171.1	(5.6)	158.0***	(5.1)
Weight	58.2	(10.4)	63.1	(9.6)	51.3***	(7.0)
BMI	21.1	(2.8)	21.6	(3.0)	20.5***	(2.5)
TCI personality traits						
Novelty Seeking (NS)	10.3	(3.2)	10.4	(3.2)	10.2**	(3.3)
Harm Avoidance (HA)	12.0	(4.1)	12.1	(4.1)	11.9*	(4.1)
Reward Dependence (RD)	10.2	(2.6)	9.7	(2.6)	10.9***	(2.5)
Persistence (P)	2.7	(1.5)	2.6	(1.5)	2.8***	(1.5)
Self Directedness (SD)	15.2	(4.4)	14.6	(4.4)	16.1***	(4.3)
Cooperativeness (C)	18.2	(3.4)	17.6	(3.4)	19.1***	(3.1)
Self Transcendence (ST)	4.1	(2.8)	4.0	(2.8)	4.2**	(2.8)

Information was expressed the total number of participants, both genders, and the mean (standard deviation) of the age, height, weight, BMI, and TCI (Temperament and Character Inventory) personality traits in first year. TCI is an instrument composed of the temperament properties and character traits. The former are four dimensions of Novelty Seeking (NS), Harm avoidance (HA), Reward Dependence (RD), Persistence (P), and the latter are three dimensions of Self Directedness (SD), Cooperativeness (C), Self Transcendence (ST). It has seven dimensions in total. The information were analyzed by Student’s t test: Male vs. Female.

***p < .001,

**p < .01,

*p < .05.

Based on the patterns of BMI changes from the first year to the fourth year, the participants were classified into seven groups: 528 students in the TT group, 240 in the NT group, 3,699 in the NN group, 116 in the NO group, and 305 in the OO group. The means (standard deviations) of their raw scores (see Tables 2 and 3) and *t*-scores (see Tables 4 and 5) were calculated. Significant sex differences were found in TCI personality traits. Therefore, we conducted ANOVAs separately for men and women to compare these groups in terms of raw data and *t*-scores of each TCI personality trait.

**Table 2:** TCI personality traits in males.

	Thinness-to-Thinness group (TT)	Thinness-to-Normal group (TN)	Normal-to-Thinness group (NT)	Normal-to-Normal group (NN)	Normal-to-Obesity group (NO)	Obesity-to-Normal group (ON)	Obesity-to-Obesity group (OO)
Number	226	117	113	2189	124	113	236
BMI in the first year	17.4*** (0.8)	17.9*** (0.5)	19.4*** (0.9)	21.2 (1.6)	23.6*** (1.2)	26.4*** (1.3)	28.3*** (3.0)
BMI change after 3 years	0.1 (0.6)	1.5*** (0.9)	-1.3*** (1.0)	0.0 (1.2)	2.4*** (1.5)	-3.1*** (1.9)	0.2 (2.2)
TCI personality traits							
Novelty Seeking (NS)	10.1 (3.1)	10.1 (3.5)	10.1 (3.3)	10.4 (3.2)	11.1 (3.3)	10.3 (3.4)	10.5 (2.9)
Harm Avoidance (HA)	12.8 (4.1)	13.2* (3.7)	11.7 (4.2)	12.0 (4.1)	12.4 (4.1)	12.5 (4.4)	12.0 (4.1)
Reward Dependence (RD)	9.3* (2.4)	9.7 (2.6)	9.4 (2.4)	9.8 (2.6)	9.7 (2.9)	9.8 (2.5)	9.5 (2.5)
Persistence (P)	2.4 (1.5)	2.7 (1.4)	2.6 (1.5)	2.6 (1.5)	2.4 (1.5)	2.6 (1.3)	2.5 (1.5)
Self Directedness (SD)	14.6 (4.4)	14.5 (4.0)	14.5 (4.7)	14.6 (4.3)	13.6 (4.8)	14.4 (4.6)	14.6 (4.4)
Cooperativeness (C)	17.2 (3.7)	17.3 (3.9)	17.5 (3.2)	17.7 (3.4)	17.3 (3.5)	17.5 (3.0)	17.6 (3.7)
Self Transcendence (ST)	4.2 (3.0)	3.8 (2.9)	3.9 (2.5)	3.9 (2.8)	4.0 (2.8)	4.1 (3.0)	4.2 (2.9)

Information are expressed the number of male participants and the mean (standard deviation) of BMI in first-year, BMI change after 3 years, and TCI (Temperament and Character Inventory) personality traits. We defined thinness as BMI < 18.5 kg/m2, normal as 18.5kg/m2 ≤ BMI < 25kg/ m2, and obesity as BMI ≥ 25 kg/m2 based on the criteria by the Guidelines for the Management of Obesity Disease 2016 [23]. Based on these three categories of BMI level (i.e., normal, obesity, and thinness), the participants were classified into seven groups depending on how their status on these categories changed from the first year to the fourth year. The seven groups were the Thinness-to-Thinness (TT), Thinness-to-Normal (TN), Normal-to-Thinness (NT), Normal-to-Normal (NN), Normal-to-Obesity (NO), Obesity-to-Normal (ON), and Obesity-to-Obesity (OO) groups. TCI is an instrument composed of the temperament properties and character traits. The former are four dimensions of Novelty Seeking (NS), Harm avoidance (HA), Reward Dependence (RD), Persistence (P), and the latter are three dimensions of Self Directedness (SD), Cooperativeness (C), Self Transcendence (ST). It has seven dimensions in total. The information was analyzed by multiple comparison of Dunnett: each group vs. the NN group.

***p < .001,

*p < .05.

**Table 3:** TCI personality traits in females.

	Thinness-to-Thinness group (TT)	Thinness-to-Normal group (TN)	Normal-to-Thinness group (NT)	Normal-to-Normal group (NN)	Normal-to-Obesity group (NO)	Obesity-to-Normal group (ON)	Obesity-to-Obesity group (OO)
Number	302	128	127	1510	42	44	69
BMI in the first year	17.4*** (0.8)	18.0*** (0.5)	19.3*** (0.8)	20.9 (1.5)	23.3*** (1.3)	26.2*** (1.4)	27.6*** (2.5)
BMI change after 3 years	-0.1 (0.8)	1.3*** (0.9)	-1.5*** (1.0)	-0.1 (1.2)	2.2*** (1.6)	-2.8*** (1.7)	0.1 (2.2)
TCI personality traits							
Novelty Seeking (NS)	9.6* (3.1)	10.2 (3.4)	9.6 (3.1)	10.3 (3.3)	10.6 (3.4)	9.8 (3.6)	10.0 (3.2)
Harm Avoidance (HA)	12.1 (4.2)	12.3 (4.2)	12.9* (3.9)	11.7 (4.1)	11.7 (4.3)	11.9 (4.5)	11.8 (3.9)
Reward Dependence (RD)	10.8 (2.6)	11.3 (1.9)	10.7 (2.5)	11.0 (2.5)	11.5 (2.2)	10.5 (3.0)	10.9 (2.4)
Persistence (P)	2.9 (1.5)	2.7 (1.6)	2.8 (1.5)	2.8 (1.5)	2.5 (1.6)	3.3 (1.4)	2.4 (1.4)
Self Directedness (SD)	16.5 (4.1)	16.4 (4.4)	15.7 (4.2)	16.1 (4.3)	14.9 (5.1)	16.0 (5.4)	15.9 (4.3)
Cooperativeness (C)	18.9 (3.3)	19.0 (3.1)	18.9 (2.8)	19.1 (3.1)	18.9 (2.9)	18.7 (3.4)	19.0 (3.2)
Self Transcendence (ST)	4.0 (2.7)	3.9 (2.8)	4.1 (3.0)	4.3 (2.8)	4.5 (3.3)	4.5 (2.8)	4.1 (2.8)

Information are expressed the number of female participants and the mean (standard deviation) of BMI in first-year, BMI change after 3 years, and TCI (Temperament and Character Inventory) personality traits. We defined thinness as BMI < 18.5 kg/m2, normal as 18.5kg/m2 ≤ BMI < 25kg/ m2, and obesity as BMI ≥ 25 kg/m2 based on the criteria by the Guidelines for the Management of Obesity Disease 2016 [23]. Based on these three categories of BMI level (i.e., normal, obesity, and thinness), the participants were classified into seven groups depending on how their status on these categories changed from the first year to the fourth year. The seven groups were the Thinness-to-Thinness (TT), Thinness-to-Normal (TN), Normal-to-Thinness (NT), Normal-to-Normal (NN), Normal-to-Obesity (NO), Obesity-to-Normal (ON), and Obesity-to-Obesity (OO) groups.

TCI is an instrument composed of the temperament properties and character traits. The former are four dimensions of Novelty Seeking (NS), Harm avoidance (HA), Reward Dependence (RD), Persistence (P), and the latter are three dimensions of Self Directedness (SD), Cooperativeness (C), Self Transcendence (ST). It has seven dimensions in total. The information was analyzed by multiple comparison of Dunnett: each group vs. the NN group.

***p < .001,

*p < .05.

**Table 4:** T scores of TCI personality traits in males.

	Thinness-to-Thinness group (TT)	Thinness-to-Normal group (TN)	Normal-to-Thinness group (NT)	Normal-to-Normal group (NN)	Normal-to-Obesity group (NO)	Obesity-to-Normal group (ON)	Obesity-to-Obesity group (OO)
Number	226	117	113	2189	124	113	236
TCI Personality traits							
Novelty Seeking (NS)	49.0 (9.8)	49.1 (10.9)	49.0 (10.4)	50.0 (10.0)	52.3 (10.2)	49.7 (10.8)	50.2 (9.2)
Harm Avoidance (HA)	51.7 (9.9)	52.8* (9.1)	48.9 (10.1)	49.8 (10.1)	50.7 (10.1)	51.0 (10.8)	49.7 (10.1)
Reward Dependence (RD)	48.6* (9.4)	50.0 (10.0)	48.7 (9.4)	50.5 (9.8)	50.0 (11.1)	50.5 (9.7)	49.1 (9.7)
Persistence (P)	48.7 (9.8)	50.4 (9.1)	49.7 (9.8)	49.9 (9.9)	48.6 (9.7)	50.1 (8.7)	49.3 (9.8)
Self Directedness (SD)	50.0 (10.0)	49.8 (9.0)	49.9 (10.7)	50.1 (9.9)	47.8 (10.9)	49.5 (10.6)	50.0 (10.0)
Cooperativeness (C)	48.8 (10.8)	49.0 (11.6)	49.6 (9.4)	50.3 (9.9)	49.2 (10.3)	49.7 (8.7)	50.0 (10.9)
Self Transcendence (ST)	50.7 (10.9)	49.2 (10.5)	49.8 (9.0)	49.7 (9.9)	49.8 (10.0)	50.3 (10.6)	50.9 (10.4)

Information are expressed the number of male participants and the mean (standard deviation) of T scores of TCI (Temperament and Character Inventory) personality traits.

We defined thinness as BMI < 18.5 kg/m2, normal as 18.5kg/m2 ≤ BMI < 25kg/ m2, and obesity as BMI ≥ 25 kg/m2 based on the criteria by the Guidelines for the Management of Obesity Disease 2016 [23]. Based on these three categories of BMI level (i.e., normal, obesity, and thinness), the participants were classified into seven groups depending on how their status on these categories changed from the first year to the fourth year. The seven groups were the Thinness-to-Thinness (TT), Thinness-to-Normal (TN), Normal-to-Thinness (NT), Normal-to-Normal (NN), Normal-to-Obesity (NO), Obesity-to-Normal (ON), and Obesity-to-Obesity (OO) groups.

TCI is an instrument composed of the temperament properties and character traits. The former are four dimensions of Novelty Seeking (NS), Harm avoidance (HA), Reward Dependence (RD), Persistence (P), and the latter are three dimensions of Self Directedness (SD), Cooperativeness (C), Self Transcendence (ST). It has seven dimensions in total.

The information was analyzed by multiple comparison of Dunnett: each group vs. the NN group.

*p < .05.

**Table 5:** T scores of TCI personality traits in females.

	Thinness-to-Thinness group (TT)	Thinness-to-Normal group (TN)	Normal-to-Thinness group (NT)	Normal-to-Normal group (NN)	Normal-to-Obesity group (NO)	Obesity-to-Normal group (ON)	Obesity-to-Obesity group (OO)
Number	302	128	127	1510	42	44	69
TCI personality traits							
Novelty Seeking (NS)	48.3* (9.3)	50.0 (10.4)	48.1 (9.8)	50.3 (10.0)	51.3 (10.3)	48.9 (11.0)	49.4 (9.8)
Harm Avoidance (HA)	50.4 (10.3)	51.1 (10.1)	52.5* (9.5)	49.6 (10.1)	49.5 (10.4)	50.1 (11.0)	49.8 (9.6)
Reward Dependence (RD)	49.6 (10.3)	51.4 (7.7)	49.2 (10.0)	50.2 (9.8)	52.2 (8.8)	48.2 (12.1)	50.2 (9.5)
Persistence (P)	50.5 (10.1)	49.5 (10.3)	49.7 (9.8)	49.9 (9.8)	48.0 (10.7)	53.0 (9.2)	47.6 (9.2)
Self Directedness (SD)	50.9 (9.5)	50.7 (10.2)	49.1 (9.8)	50.0 (10.0)	47.2 (11.8)	49.8 (12.6)	49.6 (10.1)
Cooperativeness (C)	49.5 (10.6)	49.8 (10.1)	49.4 (9.0)	50.1 (10.1)	49.2 (9.4)	48.6 (10.9)	49.7 (10.4)
Self Transcendence (ST)	49.2 (9.8)	49.0 (9.9)	49.7 (10.6)	50.2 (10.1)	50.9 (11.7)	51.1 (10.1)	49.6 (10.0)

Information are expressed the number of male participants and the mean (standard deviation) of T scores of TCI (Temperament and Character Inventory) personality traits.

We defined thinness as BMI < 18.5 kg/m2, normal as 18.5kg/m2 ≤ BMI < 25kg/ m2, and obesity as BMI ≥ 25 kg/m2 based on the criteria by the Guidelines for the Management of Obesity Disease 2016 [23]. Based on these three categories of BMI level (i.e., normal, obesity, and thinness), the participants were classified into seven groups depending on how their status on these categories changed from the first year to the fourth year. The seven groups were the Thinness-to-Thinness (TT), Thinness-to-Normal (TN), Normal-to-Thinness (NT), Normal-to-Normal (NN), Normal-to-Obesity (NO), Obesity-to-Normal (ON), and Obesity-to-Obesity (OO) groups.

TCI is an instrument composed of the temperament properties and character traits. The former are four dimensions of Novelty Seeking (NS), Harm avoidance (HA), Reward Dependence (RD), Persistence (P), and the latter are three dimensions of Self Directedness (SD), Cooperativeness (C), Self Transcendence (ST). It has seven dimensions in total.

The information was analyzed by multiple comparison of Dunnett: each group vs. the NN group.

*p < .05.

The ANOVA results showed significant differences between groups in HA (*F* = 3.076, *p* = .005) and RD (*F* = 2.428, *p* = .024) for men, and NS (*F* = 2.800, *p* = .010) for women. Then, we compared each group to the NN group using Dunnett’s multiple comparison. Both the results of the raw data (Tables 2 and 3) and *t*-scores analysis were described (Tables 4 and 5). For men, the TT group was significantly lower in RD (9.3 ± 2.4, 9.8 ± 2.6, *p* = .026), (48.6±9.4, 50.5±9.8, *p* = .026), and the TN group was significantly higher in HA (13.2 ± 3.7, 12.0 ± 4.1, *p* = .013), (52.8±9.1, 49.8±10.1, *p* = .013). For women, the TT group was significantly lower in NS (9.6 ± 3.1, 10.3 ± 3.3, *p* = .006), (48.3±9.3, 50.3±10.3, *p* = .006), and the NT group was higher in HA (12.9 ± 3.9, 11.7 ± 4.1, *p* = .011), (52.5±9.5, 49.6±10.1, *p* = .011). The NO, ON, and OO groups did not show any significant differences from the NN group in any personality trait.

## Discussion

Previous research has found that some personality traits are related to BMI. However, few longitudinal studies have been conducted, and the results contradicted each other [21]. Additionally, few studies have examined associations between personality traits and thinness among people without eating disorders. Therefore, the present study investigated associations between TCI personality traits and changes in BMI over time.

An important characteristic of this study is that the participants (i.e., Japanese university students) were classified into seven groups based on longitudinal patterns of changes in BMI status, which reflect Normal versus Obesity/Thinness distinctions, from the first year to the fourth year. As significant sex differences were found in TCI personality traits, the data of men and women were analyzed separately.

For male students, this study showed that the TT group was lower in RD than the NN group. Male students who remained thin in terms of BMI over time were less likely to seek attention or approval from others than those who remained normal. Many men desire to have a more muscular body [24–26] because of social norms that consider the ideal male form to be muscular [27]. However, a person with low RD tends to have shallow relationships with others [18]. Therefore, these men might be less susceptible to sociocultural pressure for men to be muscular, and hence, may become more indifferent to their own body shape. This might be why these students continued to be thin. Next, the TN group was higher in HA than the NN group. People with high HA tend to show pessimistic worry, anticipatory anxiety, and introversion [18]. Neuroticism is conceptually similar to HA, as both are related to a person’s tendency toward anxiety; additionally, neuroticism has been found to be positively correlated with HA [28]. Many previous studies have suggested that high neuroticism increased mortality [29] or was irrelevant [30]. On the other hand, it should not be overlooked that some studies have suggested that neuroticism may be protective against mortality. These studies have examined the possibility that certain aspects of neuroticism may be relevant as protective factors [31] or may have protective effects on health through interaction with other personality traits [32], based on Friedman’s idea of healthy neuroticism [33]. Neuroticism is positively associated with one factor of body vigilance: sensation awareness belief. Body vigilance has an inhibitory effect on the relationship between neuroticism and health behaviors, indicating that neuroticism may be associated with healthier behaviors through vigilance [34]. Therefore, those with high neuroticism may have increased vigilance and attention to their bodies due to anxiety, and may actively try to be healthy. In addition, Japanese male university students desire to be larger than their current values for ideal height, weight, BMI, and body shape [35]. Hence, the male students in the TN group may have been concerned about their current body shape and health condition, and in an attempt to attain an ideal body, they could have improved their BMI status from thin to normal.

In female students, the TT group was lower in NS than the NN group. No prior studies have directly addressed the association between thinness and NS in women. People with low NS tend to be less impulsive, indifferent, stoic, and avoid new situations and changes [17, 18]. Therefore, it could be speculated that a person with lower NS might tend to have less interest in eating and be reluctant to expend effort on food consumption. This can explain why these women continued to maintain their thinness status. Next, the NT group was higher in HA than the NN group. This result is contrary to previous research in Europe and the United States that found people high in HA tend have a high BMI [21, 36]. Additionally, a study in Japan showed that women with high HA tend to have increased BMI [37]. The researchers speculated that women of this type are more likely to have poor physical health because they tend to have negative emotional responses [37, 38]. In contrast, another study in Japan found a negative correlation between HA and BMI [39]. Similarly, studies with Japanese and Korean samples found that people with high neuroticism tended to have low BMI [40–43]. The ratio of Asian women with thinness status is notably higher than that of European and American women [44]; this might reflect a strong desire in Asian females to be thin because of media influence [45]. Those with stronger neuroticism might be even more prone to this desire to be thin in response to media influence or negative comments from others regarding their body shape, because of their sensitivity to evaluation by others [46–48]. Thus, our finding suggesting a negative relationship between HA and BMI mostly aligned with existing research focused on Asian people, but not research with European and American people.

Unlike previous research, the relationships between personality traits and obesity in the present study were not significant in both male and female students. The study also found a higher level of HA in the men whose BMI status improved from thinness to normal and in the women whose BMI status worsened from normal to thinness. The reason that the results differed between men and women as described above may be because the idealized body types are different depending on gender (i.e., this is a socio-cultural factor). In conclusion, although existing research has examined personality traits primarily in terms of their relationship with obesity, it will also be necessary to focus on thinness in future studies.

The limitations of this study include the following. First, students’ history of eating disorders was self-reported, with no positive responses. However, the number of people with eating disorders has been increasing in Japan [49]; one study reported that 1.8% of National University students were diagnosed with an eating disorder [2]. Because people tend to conceal the disease [50], it was difficult to obtain an objective history of eating disorders in this study. Second, the sample sizes of groups included in the analyses were uneven. No previous studies were found that investigated the averages of TCI personality traits (two-point scale) in a large sample of Japanese university students. Therefore, it is difficult to compare means in this research with those of national norms. We consider that further research on clinical differences is needed, such as matching the sample size. Third, when we examined associations between TCI personality traits and BMI, factors such as eating behavior, living environment, body image, body vigilance, health concerns, and interactions between TCI personality traits at both subscale and item levels were not considered. Fourth, detailed information on changes in BMI was ignored, because we changed the originally continuous data to ordered categories (i.e., obesity, normal, and thinness). For example, even if a participant only slightly increased their BMI from 24.9 kg/m^2^ in the first year to 25.1 kg/m^2^ three years later, their BMI status would have changed from normal to obesity. In contrast, even if a participant substantially reduced their BMI from 32.0 kg/m^2^ in the first year to 25.3 kg/m^2^ three years later, their BMI status of obesity would have remained the same. Fifth, a change in BMI does not necessarily mean a corresponding change in visceral fat. In the future, it will be necessary to consider increasing participants in certain subgroups, and to consider factors such as eating behaviors (including items related to eating disorders), the presence or absence of exercise habits, and whether participants live alone. Finally, although there was a statistically significant difference, since the mean differences in both raw scores and those converted to *t*-scores are clinically small, these results must be interpreted with caution, and further study is necessary. However, we believe that this study is a foothold for future developmental research in that it is the first to examine personality traits and BMI changes in a non-clinical population of Japanese university students.

## Conclusion

This is the first study to longitudinally examine the relationship between TCI personality traits and BMI changes for Japanese university students. No existing studies have examined associations between TCI personality traits and thinness without using a clinical population with eating disorders until now. The results revealed weak associations between thinness and TCI personality traits among Japanese university students. Further elaboration of the relationship between obesity or thinness and personality traits may help to provide effective preventive interventions in these areas.
